# Bioremediation: Data on *Pseudomonas aeruginosa* effects *on the bioremediation of crude oil polluted soil*

**DOI:** 10.1016/j.dib.2018.04.102

**Published:** 2018-05-03

**Authors:** Modupe Elizabeth Ojewumi, Joshua Olusegun Okeniyi, Jacob Olumuyiwa Ikotun, Elizabeth Toyin Okeniyi, Valentina Anenih Ejemen, Abimbola Patricia Idowu Popoola

**Affiliations:** a Chemical Engineering Department, Covenant University, Ota, Ogun state, Nigeria; b Mechanical Engineering Department, Covenant University, Ota, Ogun state, Nigeria; c Chemical, Metallurgical and Materials Engineering Department, Tshwane University of Technology, Pretoria, South Africa; d Department of Civil Engineering and Building, Vaal University of Technology, Vanderbijlpark, South Africa; e Petroleum Engineering Department, Covenant University, Ota, Ogun state, Nigeria

**Keywords:** Bioremediation, Onshore oil pollution simulating system, *Pseudomonas aeruginosa*, UV/VIS Spectrophotometry, Absorbance, Crude oil polluted soil

## Abstract

This data article details *Pseudomonas aeruginosa* effects on the bioremediation of soil that had been polluted by different concentrations, 5% w/w and 8% w/w, of raw (for simulating oil spills from well-heads) and treated (for simulating oil spills from flow lines/storage tanks) crude oil. UV/VIS spectrophotometry instrumentation was used for obtaining absorbance measurements from the Nigerian Escravos Light blend (sourced from Chevron® Nigeria) of crude oil polluting soil samples, which, thus, also simulates light and heavy onshore oil spillage scenarios, in a 30-day measurement design. Data on bioremediation effects of *Pseudomonas aeruginosa* added to the crude oil polluted soil samples, and which were monitored at intervals via the absorbance measurement techniques, are presented in tables with ensuing analyses for describing and validating the data presented in graphs. Information from the presented data in this article is useful to researchers, the oil industries, oil prospecting communities, governments and stakeholders involved in finding solution approach to the challenges of onshore oil spills. This information can also be used for furthering research on bioremediation kinetics such as biostimulant analyses, polluting hydrocarbon content/degradation detailing, by *Pseudomonas aeruginosa* strain of microorganism, on petroleum pollutant removal from soil that had been polluted by crude oil spillage.

**Specifications table**

**Table:** 

Subject area	*Engineering*
More specific subject area	*Chemical Engineering, Environmental Engineering, Sustainable Environmental Sciences and Management*
Type of data	*Tables, graphs, figures*
How data was acquired	*Absorbance data measurements from crude oil polluted soil sample systems using* a Jenway 6405 *ultra violet visible (UV/VIS)* spectrophotometer instrument
Data format	*Raw, statistically analyzed*
Experimental factors	*Absorbance data monitoring were executed as laboratory experimental data sourcing on crude oil polluted soil samples that had been inoculated using Pseudomonas aeruginosa strain of bacteria*
Experimental features	*Air dried loamy soil was polluted by two different concentrations of two types (raw and treated) of Nigerian Escravos Light blend of petroleum and then inoculated using Pseudomonas aeruginosa strain of microbe, for bioremediation monitoring via periodic absorbance measurements*
Data source location	*Loamy soil was collected from Covenant University Farm, Nigerian Escravos Light crude oil blend was sourced from Chevron® Nigeria, Delta State, Nigeria, Absorbance measurement procedures was carried out at Covenant University, Ota, Nigeria (Latitude 6.6718°N, Longitude 3.1581°E).*
Data accessibility	*The comprehensive dataset of the bioremediation effects of Pseudomonas aeruginosa, via absorbance monitoring, on soil that has been polluted by raw and treated crude oil is made available in this data article*

**Value of the data**

• Information in this data article is valuable for implementing remediation amendment, using the techniques of microorganism mediated oil pollutant removal (bioremediation), which is being preferred as an environmentally-friendly/sustainable approach in the literature, on soil that has been polluted by oil spillage [1], [2], [3], [4], [5].
• Data on the use of different concentrations of crude oil in the polluted soil system is useful for decision making on remediation of soil pollution that could ensue from light oil spill and from heavy oil spill situations [6], [7].
• Dataset on the performance of *Pseudomonas aeruginosa* on raw and treated Nigerian Escravos Light crude oil as soil pollutant exhibits the potential of detailing its effectiveness and promoting the usage of this microbial strain as an economical and efficient bioremediation technique [1], [8].
• Absorbance data from the *Pseudomonas aeruginosa* inoculation in crude oil polluted soil, from this data article, can be employed for detailing bioremediation kinetic, such as petroleum hydrocarbon degradation potential and parameters, by this strain of bacteria on the Nigeria Escravos light crude oil polluted soil and such information will be useful to stakeholders involved in oil polluted soil amendment [1], [8].
• Presented analyses of bioremediation data in this article is valuable for describing, analyzing, validating and detailing reliability of measured data [9], [10], [11], [12], [13], [14], [15], which for the present case involves dataset of bioremediation effects investigations and implementations. This could foster repeatability and/or applications of the analytical methods for future works that could range from laboratory experiment, pilot scale up to real-time field executions of crude oil polluted soil amendments.

## 1 Data

The always increasing global energy demand makes it imperative that the world is still highly dependent on petroleum products for meeting energy needs in many ramifications of livelihood, a condition that the necessitates continuous extraction/production of petroleum from its location deep down the earth [2], [16], [17], [18], [19], [20]. The situation ensuing from this include crude petroleum oil spill that could be through uncontained excessive pressure from production installations/platforms, e.g. raw crude oil from well-heads, blowouts, etc., or from transportation or improper handling e.g. of treated crude oil in flow lines or storage tanks [20], [21]. The resulting oil spill that could be into marine (offshore) or soil (onshore) environments are very toxic and hazardous to the environmental ecosystem and could adversely affect well-being of living organs, air, water and soil processes as well as the potential of fire hazards [22], [23], [24]. Onshore spill of crude oil affects healthy living in the society, agricultural productivity, groundwater/sources for potable water, and living biota in flowing streams/rivers, among others [5], [25], [26], [27]. Avoiding or mitigating these adverse effects from crude oil spillage situation necessitates needs for amending the soil via the procedure known as remediation.

Among known methods for remediating crude oil polluted soil, including physical separation, chemical degradation, photodegradation and bioremediation, the method of bioremediation is attracting preference due to its comparative effectiveness, relatively low cost and eco-friendliness compare to other the techniques [1], [2]. Unlike bioremediation, other methods that could be used for oil polluted soil remediation have also been recognized with the potential of leaving daughter compounds, i.e. secondary residuals, after the parent/primary crude oil pollutant has been removed, which can even exhibit higher toxicity levels than the parent crude oil pollutant [1], [2]. In contrasts, bioremediation technique usage detoxifies contaminants in crude oil and effectively removes pollutant by destroying them in the stead of transferring them to other medium [2], [3], [4].

Studies have employed plants species for bioremediation, in processed known as phytoremediation [7], but the use of microorganisms as biologically-mediated remediation of crude oil polluted soil is still linked to the effectiveness of phytoremediation systems. This is due to the fact that microorganisms are still required in the rhizosphere of plants for efficient crude oil polluted soil remediation via phytoremediation [6], [23]. This is making the use of microorganism for crude oil polluted soil remediation purposes of increasing interests to researchers and stakeholders involved in crude oil polluted soil amendment. Bacteria strains of microbes, including *Pseudomonas aeruginosa*, have been used in reported works for effective repair of crude oil polluted soil [20], [28]. However, there is paucity of reported work employing *Pseudomonas aeruginosa* for the bioremediation of Escravos Light crude oil blend obtainable in Nigeria. No dataset of absorbance measurements exists in the literature from the *Pseudomonas aeruginosa* effects on raw and treated Escravos Light blend of crude oil polluted soil systems. This data article, therefore, presents absorbance dataset and its analyses obtained from two different concentrations, for simulating light and heavy onshore spill, of raw and treated Escravos Light crude oil polluting soil systems that was inoculated for bioremediation effect using *Pseudomonas aeruginosa*.

Table 1, therefore, presents absorbance data measurements obtained from raw and treated types of Escravos Light crude oil polluted soil that had been inoculated with *Pseudomonas aeruginosa* strain of microorganism for bioremediation effects. Shown in the table are the absorbance data for 5% w/w concentration of crude oil pollutant in soil, for simulating light oil spill, as well as the data for 8% w/w soil-polluting crude oil concentration, for simulating the spillage of heavy oil. These raw data measurements are in duplicate measures of experimental design, taken in five days interval for the first 20 days and in 10 days interval, thereafter, for making up the 30-day period of absorbance data measurements from the crude oil polluted soil. This later jump in experimental monitoring interval was done for noting whether there will be a significant persistency of bioremediation effect by the *Pseudomonas aeruginosa* strain of micro-organism on the different types and concentrations of crude oil polluted soil systems, or otherwise. For these reasons, therefore, the table also includes the average of the periodic absorbance measurements taken in the intervals of measuring *Pseudomonas aeruginosa* effects on the different types and concentrations of crude oil polluted soil systems.

**Table 1:** Absorbance data of *Pseudomonas aeruginosa* effects on Escravos Light crude oil polluted soil.

Type of crude oil pollutant in soil	Time (day)	**5% w/w**			**8% w/w**		
		Absorbance (nm)	Absorbance {Duplicate} (nm)	Periodic Average Absorbance (nm)	Absorbance (nm)	Absorbance {Duplicate} (nm)	Periodic Average Absorbance (nm)
Raw Crude Oil Polluted Soil (**RCOP**)	0	0.365	0.36	0.3625	0.409	0.405	0.407
	5	0.423	0.417	0.42	1.177	1.177	1.177
	10	0.289	0.312	0.3005	0.247	0.239	0.243
	15	0.197	0.195	0.196	0.134	0.139	0.1365
	20	0.139	0.139	0.139	0.088	0.088	0.088
	30	0.034	0.034	0.034	0.078	0.08	0.079
Treated Crude Oil Polluted Soil (**TCOP**)	0	0.105	0.104	0.1045	0.253	0.253	0.253
	5	0.085	0.092	0.0885	0.112	0.117	0.1145
	10	0.027	0.026	0.0265	0.053	0.053	0.053
	15	0.0187	0.019	0.01885	0.026	0.0266	0.0263
	20	0.013	0.013	0.013	0.007	0.007	0.007
	30	0.009	0.009	0.009	0.006	0.006	0.006

**Fig. 1:**
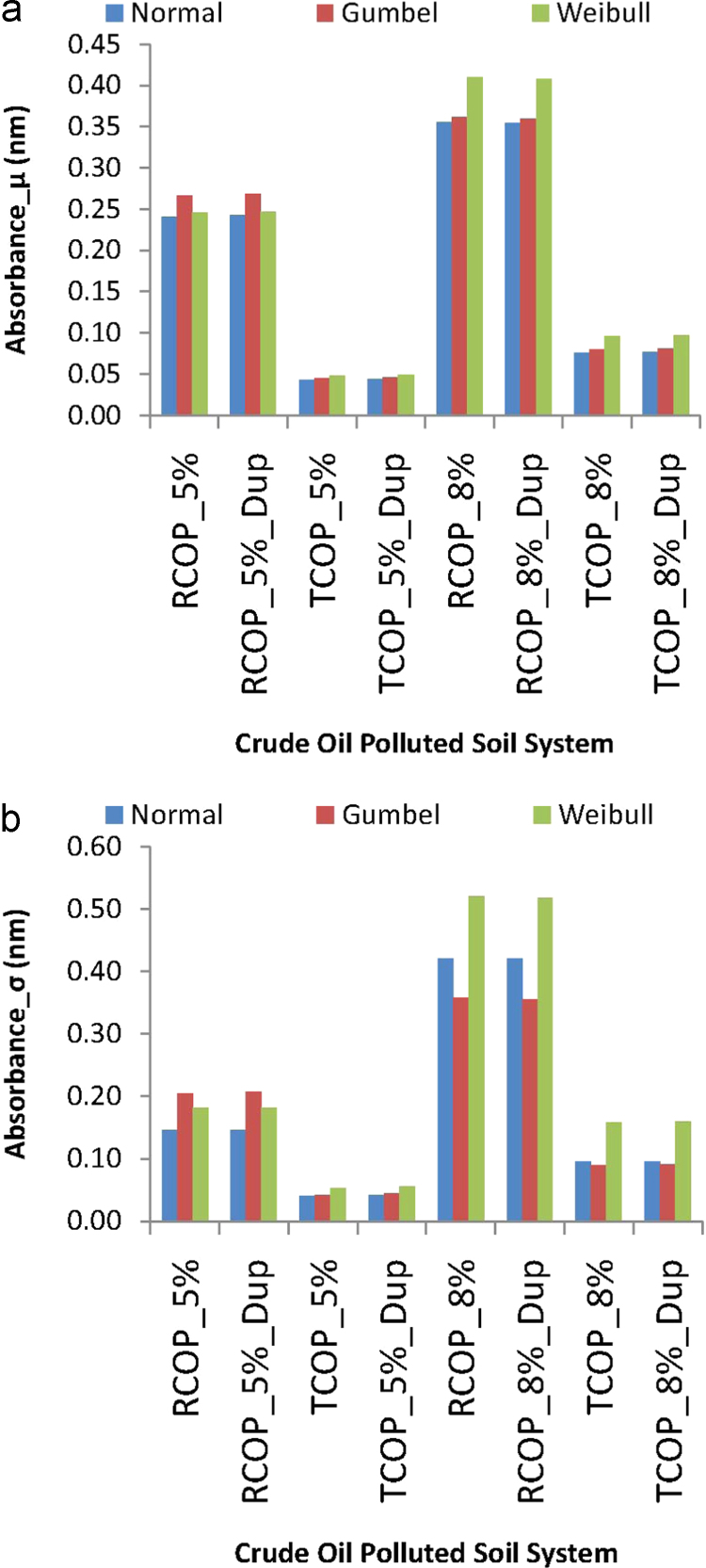
The Normal, Gumbel and Weibull descriptive statistics models of raw duplicated measurements of absorbance data from crude oil polluted soil inoculated with *Pseudomonas aeruginosa* (a) mean absorbance (b) standard deviation of absorbance.

For aiding further analyses of the data proceeding from Table 1. Fig. 1 presents plots of the descriptive statistics of the duplicated raw measurements of absorbance data by the Normal, Gumbel and Weibull probability distribution modeling functions. The use of these three distribution fitting models will lend insight into whether the bioremediation data could be best described by the random sampling distribution of the Normal and/or of the Gumbel and/or of the Weibull probability density modeling functions. For specific instance and comparison of the probability fitting models, the Normal distribution is a general descriptive statistics model that exhibits the advantage of being the simplest probability distribution that could be applied to randomly distributed data. This simplicity of application of the Normal distribution follows from the fact that the mathematical relationships for estimating important parameters for the distribution model are well-known and easily computed [29]. In contrast, the Gumbel and the Weibull distributions are extreme value distribution models useful for studying the existence of asymptotic test-response in the data that could motivate underlying extreme value process in the *Pseudomonas aeruginosa* bioremediation effects on the different types and concentrations of crude oil polluted soil systems. From these two models, the Gumbel distribution is the extreme value distribution of maxima, which indicates whether the maximum of the tested effect in a system is responsible for the reliability or the hazard encountered in the system. The Weibull distribution is the extreme value distribution of minima, which details whether the minimum of the tested effect exhibits responsibility for the reliability or the hazard in the test-system. However, all of these distribution modeling tools suffer the disadvantage that their usage for describing data not distributed like the distribution could lead to grossly erroneous conclusion [30].

**Fig. 2:**
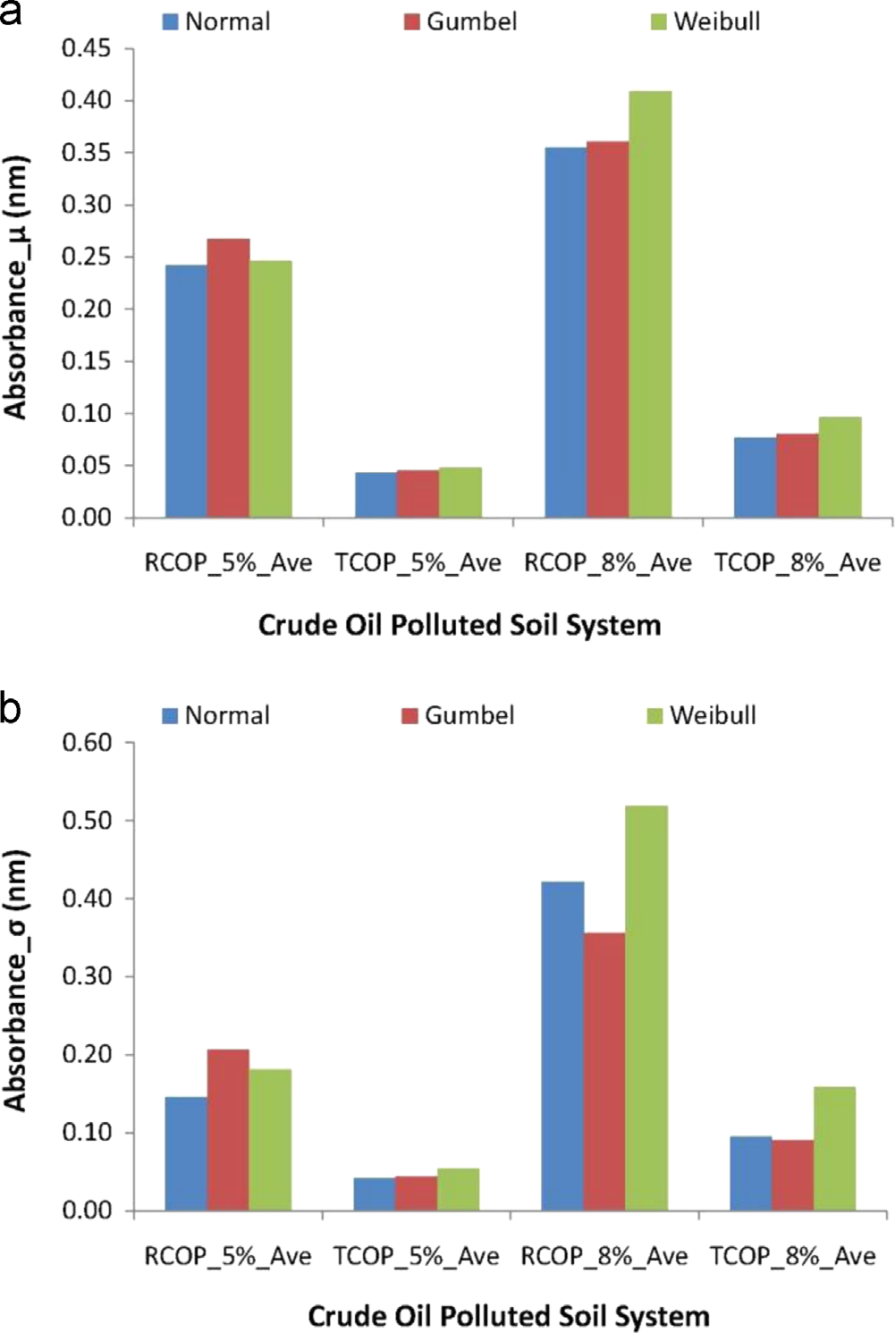
The Normal, Gumbel and Weibull descriptive statistics models of periodically averaged measurement of absorbance data from crude oil polluted soil inoculated with *Pseudomonas aeruginosa* (a) mean absorbance (b) standard deviation of absorbance.

Thus, Fig. 1 entails the plot of mean (μ) models of absorbance data by these statistical distributions in Fig. 1(a) and the standard deviation (*σ*) models of absorbance data by the distributions in Fig. 1(b). In the figure, RCOP refers to the raw crude oil polluted soil system, and TCOP refers to the treated oil polluted soil system, while the duplicate sampling was indicated by attaching the tag “_Dup”. It is also worth noting that the mean and standard deviation modeling in Fig. 1 employ the maximum likelihood estimation procedures [29], [31], [32], [33], [34], [35] for these measurements of central tendencies and measurements of dispersions using the Normal, the Gumbel and the Weibull distribution modeling. From a similarly considerations, therefore Fig. 2 presents plots of these descriptive statistics applications to the evaluated averaged data obtained from the duplicates of absorbance periodic measurements, and for these, also, the mean models are in Fig. 2(a) while the standard deviation models are in Fig. 2(b). In this second figure, the delineating tags now include “_5%” and “_8%” for indicating the 5% w/w and the 8% w/w concentrations of crude oil pollutant in the soil sample systems, as well as “_ave” was used for indicating the periodic average of absorbance measurements.

## 2 Experimental design, materials and methods

For the measured data in this article, loamy soil was collected from Covenant University Farm. This soil from the agricultural site was air dried before being polluted with two different pollution concentrations, i.e. 5% and 8% w/w, of raw and treated Escravos Light crude oil blend obtained from Chevron® Nigeria Limited, Delta State, Nigeria. This was followed by the inoculation of each crude oil polluted soil design with *Pseudomons aeruginasa*, a bacteria strain of microorganism, which was collected from the Applied Biology and Biotechnology Unit of the Department of Biological Sciences, Covenant University, Ota, Ogun State, Nigeria [36], [37]. The *Pseudomons aeruginasa* bacteria strain usage for inoculation of the crude oil polluted soil system was at the concentration of 0.05 v/v of the microbial strain (obtained from Mueller Hilton Broth suspension) to each of the crude oil polluted soil systems for the study. From each of the systems of crude oil polluted soil detailed, selected mass sample was taken and dissolved in hexane by stirring in a magnetic stirrer. A portion from this dissolution was measured and made up with *n*-hexane for determination of absorbance at wavelength of 420 nm via a Jenway 6405 UV/VIS Spectrophotometer. These absorbance measurement experiments were executed in duplicates, starting from the 0th day, then in five days interval for the first 20 days and, thereafter, in 10 days interval, for making up the 30-day experimental design system (as earlier detailed), from which the data, presented in Table 1, was obtained.

**Fig. 3:**
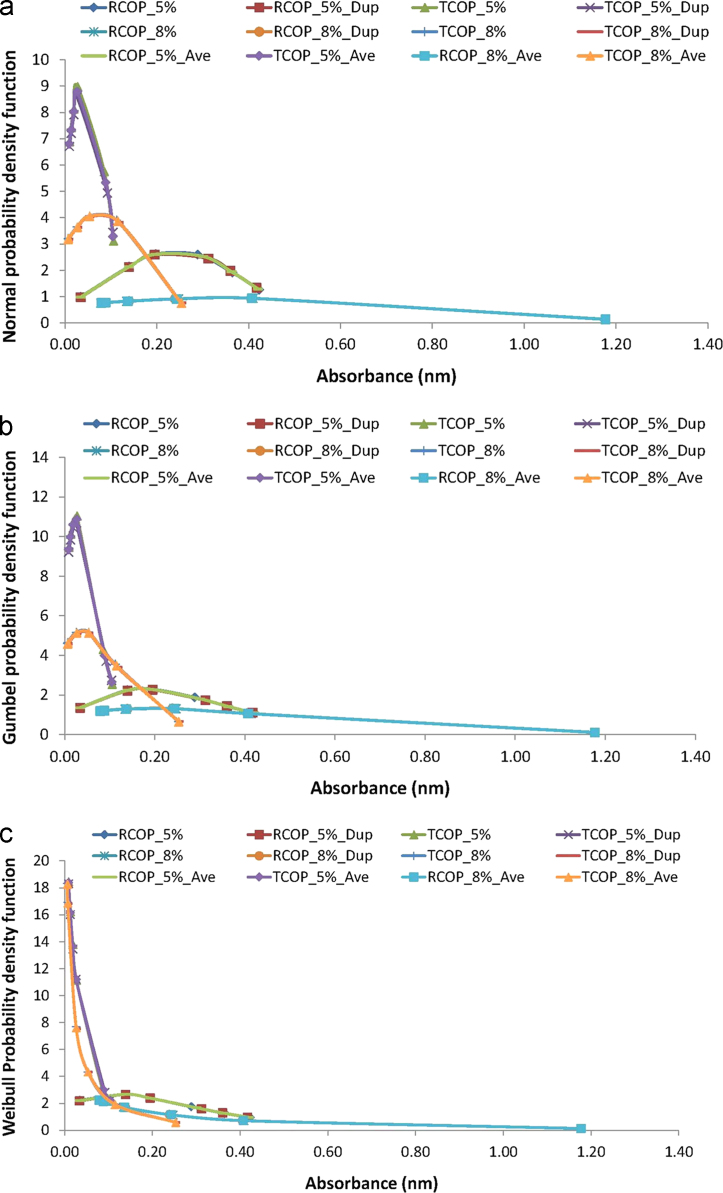
Fittings of absorbance data from the crude oil polluted soil systems to the probability distribution functions of the (a) Normal (b) Gumbel, and (c) Weibull.

The descriptive statistics of the absorbance data from the crude oil polluted soil systems inoculated with *Pseudomonas aeruginosa*, as were presented in Fig. 1 and in Fig. 2, employ the distribution fittings of the Normal, the Gumbel and the Weibull probability density models [38], [39], [40], [41], [42]. These fittings of the absorbance data to the each of the probability distribution functions are respectively presented in Fig. 3, i.e. for the Normal distribution in Fig. 3(a), the Gumbel distribution in Fig. 3(b), and the Weibull distribution in Fig. 3(c).

**Fig. 4:**
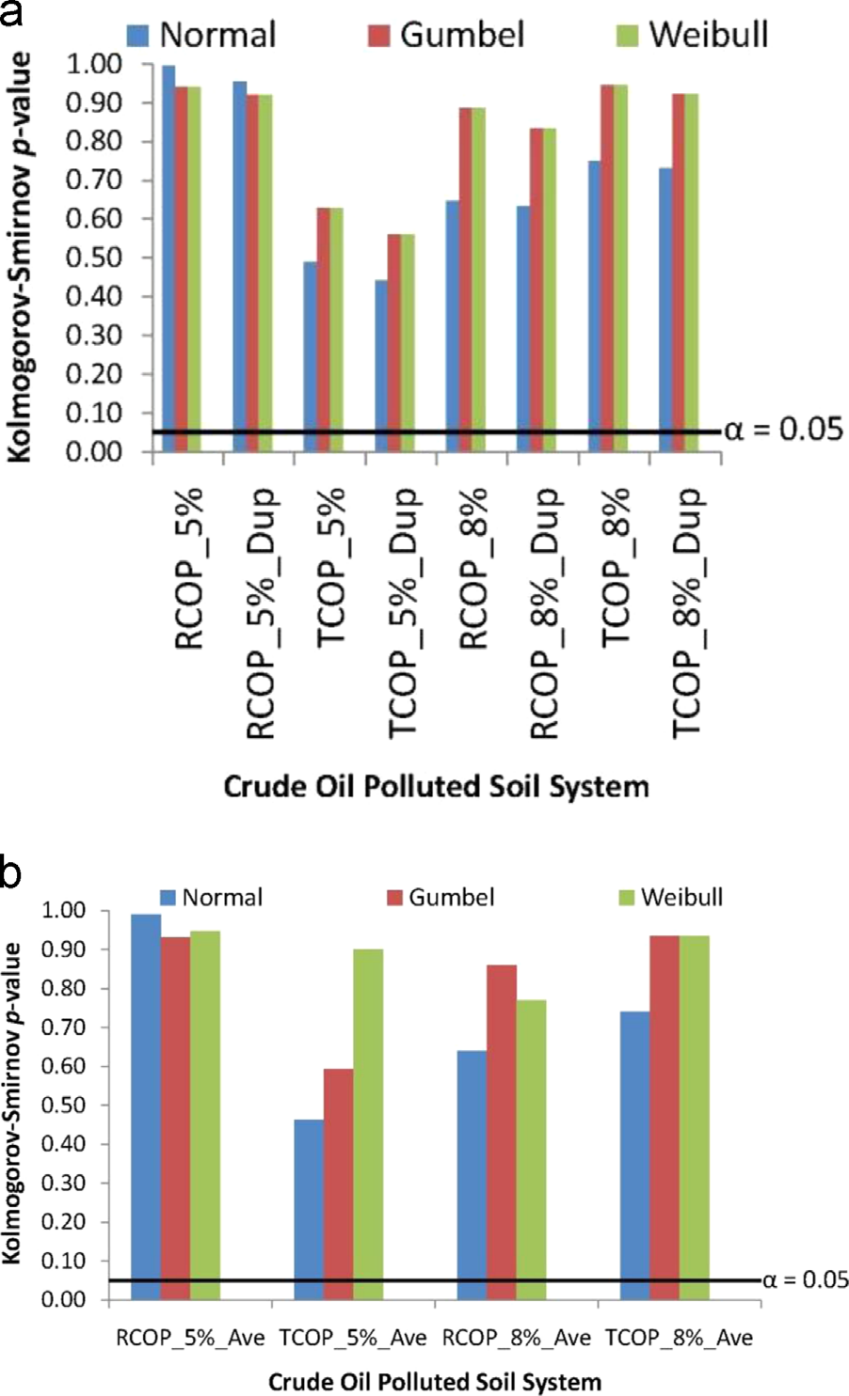
Compatibility testing to the Normal, Gumbel and Weibull distributions via the Kolmogorov–Smirnov goodness-of-fit statistics (a) absorbance data experimentally measured from crude oil polluted soil having *Pseudomonas aeruginosa* inoculants (b) periodically averaged absorbance data from the duplicates of polluted soil system.

Compatibility of the absorbance data, from the crude oil polluted soil systems having *Pseudomonas aeruginosa* inoculants, to the fittings of each of the Normal, the Gumbel and the Weibull probability distributions requires the Kolmogorov–Smirnov goodness-of-fit test-statistics, *α*=0.05 significant level [43], [44], [45], [46], [47]. This Kolmogorov–Smirnov goodness-of-fit testing of compatibility statistics application to the absorbance data in this article are presented in graphical plots in Fig. 4, which also shows the linear plot of *α*=0.05 level of significance. By these, therefore, plots of Kolmogorov–Smirnov goodness-of-fit probability value (*p*-value) that does not attain the *α*=0.05 linear plot in Fig. 4 indicate data that are not distributed like the probability distribution being applied for describing the model. In contrast, plots of Kolmogorov–Smirnov *p*-value that overshot the *α*=0.05 linear plot in Fig. 4 are indicative of data that are distributed like the probability distribution of application to the model.

**Fig. 5:**
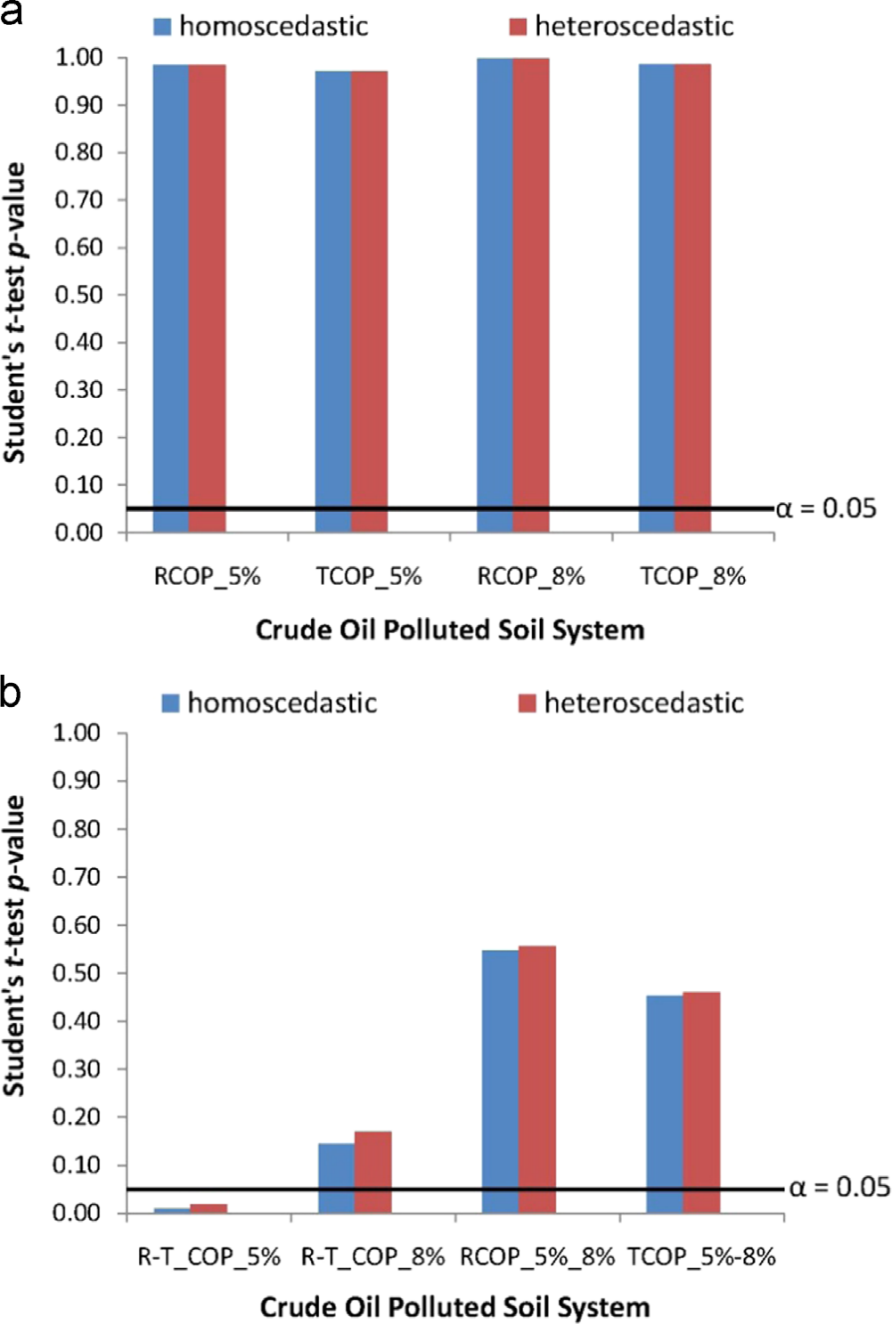
Student's *t*-test statistics of significance of differences in absorbance data from the *Pseudomonas aeruginosa* inoculated crude oil polluted soil systems (a) between-duplicate tests-of-significance (b) between different crude oil concentration/different crude oil types tests-of-significance.

The duplicated design of absorbance measurements, as well as the different designs of crude oil pollutant systems in the soil samples, necessitates testing significance of differences from the measured absorbance data. For these, the between-duplicate and the between-different crude oil/soil system pollution test of significance models, employing the Student's *t*-test statistics was applied to the absorbance data using the homeoscedastic (equal variance) and the heteroscedastic (unequal variance) assumption models [9], [11], [42], [48], [49], [50]. Fig. 5, therefore shows plots of the Student's *t*-test statistics application to the absorbance data from the crude oil polluted soil system having *Pseudomonas aeruginosa* inoculants. In the figure, Fig. 5(a) shows the between-duplicate and Fig. 5(b) shows the between-different crude oil/soil pollution system tests of significance. Also included in each of Fig. 5(a) and (b) are linear plots of *α*=0.05, for which Student's *t*-test *p*-value not attaining the *α*=0.05 linear plot is indicative of the fact that the experimentally observed differences between the two datasets being compared are statistically significant. Otherwise, Student's *t*-test *p*-value that overshot the *α*=0.05 linear plot indicates that the experimentally observed differences between the two datasets being compared are statistically not significant, but are due to randomization ensuing from the experimental test-measurements.

Worth noting includes the fact that the tags of abbreviations employed in Fig. 5(a) could be detailed as:

• RCOP_5%: compares significance of differences between datasets from the duplicated sampling for raw crude oil polluted soil system having 5% w/w crude oil/soil pollution concentration;
• TCOP_5%: compares significance of differences between datasets from the duplicated sampling for treated crude oil polluted soil system having 5% w/w crude oil/soil pollution concentration;
• RCOP_8%: compares significance of differences between datasets from the duplicated sampling for raw crude oil polluted soil system having 8% w/w crude oil/soil pollution concentration;
• TCOP_8%: compares significance of differences between datasets from the duplicated sampling for treated crude oil polluted soil system having 8% w/w crude oil/soil pollution concentration.

Also, the tags of abbreviation used in Fig. 5(b) are as follows:

• R-T_COP_5%: compares significance of differences between dataset from raw and dataset from treated crude oil polluted soil systems having 5% w/w crude oil/soil pollution concentration;
• R-T_COP_8%: compares significance of differences between dataset from raw and dataset from treated crude oil polluted soil system having 8% w/w crude oil/soil pollution concentration;
• RCOP_5%_8%: compares significance of differences between dataset from the soil systems polluted with 5% w/w and dataset from the soil systems polluted with 8% w/w raw crude oil pollutant;
• TCOP_5–8%: compares significance of differences between dataset from the soil system polluted with 5% w/w and dataset from the soil systems polluted with 8% w/w treated crude oil pollutant.

**Fig. 6:**
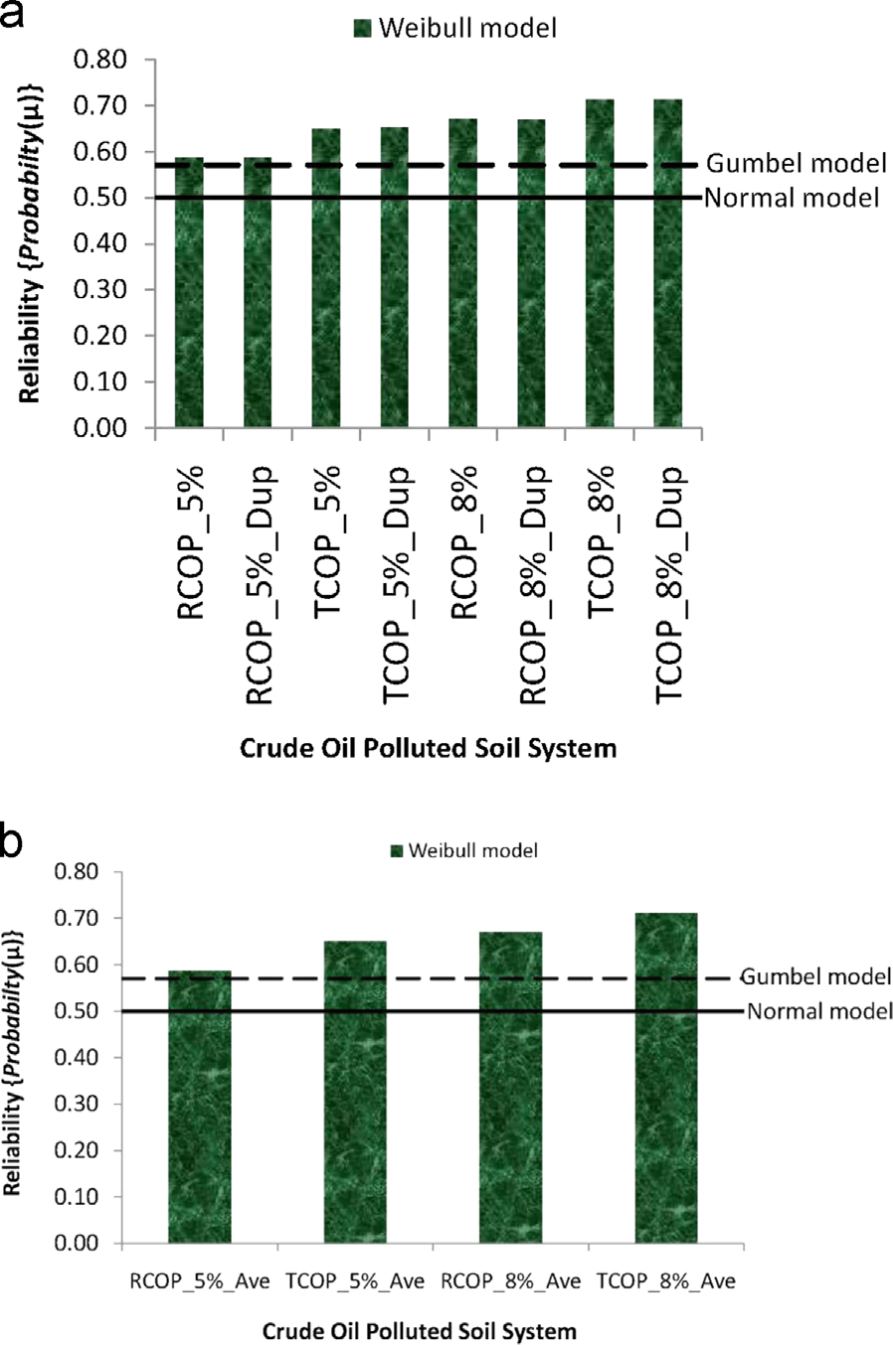
Reliability plots of absorbance data from the *Pseudomonas aeruginosa* inoculated crude oil polluted soil systems (a) reliability from measured duplicates absorbance data (b) reliability from periodically averaged absorbance data.

Proceeding from the probability distributions, employed in this data article, is the measurement of the probability of obtaining the analyzed mean of the raw absorbance data measurements, Fig. 1(a), and of the periodically averaged absorbance, Fig. 2(a), from the crude oil polluted soil systems. This particular measure of probability indicates the reliability of either the raw or the periodically averaged data on the remediation effect of *Pseudomonas aeruginosa* in the different concentrations/types of crude oil polluted soil systems. Though, it is worth noting, that the reliability (or the probability of obtaining the mean) monotonically=0.5 via the Normal, or=0.5704 via the Gumbel distribution models, irrespective of the mean value, the value of this parameter varies with the mean values in the Weibull model [39], [51], [52]. This variability of reliability from the Weibull probability distribution modeling, therefore, aids comparisons with the reliability obtained from the other two distribution function models, of the Normal and the Gumbel. Thus, Fig. 6 presents the plots of the reliability by the Weibull probability distribution modeling of the absorbance data, for the raw in Fig. 6(a) and the periodically averaged measurements in Fig. 6(b). In the figure, also, the monotonic reliability value of 0.5 from the Normal and of 0.5704 from the Gumbel distributions are shown as linear plots. These reliability values are indicative of the cumulative distribution function applications of the Normal, the Gumbel and the Weibull to the mean models of these distribution fitting functions. They exhibit the significance that the estimated values, as indicated in Fig. 6, detailed values that could be related to the degrees of the bioremediation effect by the *Pseudomonas aeruginosa* on the different crude oil polluted test-systems. The implications ensuing from the usage of such estimated model for reliability follows from the fact that for the types of experimental measurements in this study, it is desirable to at least obtain the mean value of bioremediation effect estimated for each test-system, if not more, rather than the desirable event in failure-causing data which is that of obtaining lesser value than the estimated failure-inducing mean value.
